# Synthesis, Phase Transformations and Strength Properties of Nanostructured (1 − x)ZrO_2_ − xCeO_2_ Composite Ceramics

**DOI:** 10.3390/nano12121979

**Published:** 2022-06-09

**Authors:** Askhat Berguzinov, Artem Kozlovskiy, Ainagul A. Khametova, Dmitriy I. Shlimas

**Affiliations:** 1 Department of Heat Power Engineering, Toraighyrov University, Pavlodar 140000, Kazakhstan; 2 Laboratory of Solid State Physics, The Institute of Nuclear Physics, Almaty 050032, Kazakhstan; shlimas@mail.ru; 3 Engineering Profile Laboratory, L.N. Gumilyov Eurasian National University, Nur-Sultan 010008, Kazakhstan; khametovaaa@gmail.com

**Keywords:** composite ceramics, zirconium dioxide, dislocation hardening, mechanical testing, phase transformations, materials for nuclear power

## Abstract

The aim of this work is to study the properties of nanostructured (1 − x)ZrO_2_ − xCeO_2_ composite ceramics, depending on the content of oxide components, as well as to establish the relationship between the phase composition of ceramics and strength properties. The choice of (1− x)ZrO_2_ − xCeO_2_ composite ceramics as objects of study is due to the great prospects for using them as the basis for inert matrix materials for nuclear dispersed fuel, which can replace traditional uranium fuel in high-temperature nuclear reactors. Using X-ray diffraction, it was found that the variation of the oxide components leads to phase transformations of the Monoclinic-ZrO_2_ → Monoclinic − Zr_0.98_Ce_0.02_O_2_/Tetragonal − ZrO_2_ → Tetragonal − Zr_0.85_Ce_0.15_O_2_ → Tetragonal − ZrCeO_4_/Ce_0.1_Zr_0.9_O_2_ type. As a result of mechanical tests, it was found that the formation of tetragonal phases in the structure of ceramics leads to strengthening of ceramics and an increase in crack resistance, which is due not only to an increase in the crystallinity degree, but also to the effect of dislocation hardening associated with a decrease in grain size. It has been established that a change in the phase composition due to phase transformations and displacement of the ZrO_2_ phase from the ceramic structure with its transformation into the phase of partial replacement of Zr_0.85_Ce_0.15_O_2_ or Ce_0.1_Zr_0.9_O_2_ leads to the strengthening of ceramics by more than 3.5–4 times. The results of resistance to crack formation under single compression showed that the formation of the ZrCeO_4_ phase in the structure of ceramics leads to an increase in the resistance of ceramics to cracking by more than 2.5 times.

## 1. Introduction

As it is known, one of the priority tasks in the development of nuclear energy is the development of new types of nuclear fuel, which can significantly increase the productivity of nuclear installations by increasing the degree of fuel burnup [1,2,3]. The main criteria for new types of nuclear fuel are: (1) the possibility of operation in a wide temperature range; (2) high hardness and strength of fuel cells; (3) high resistance to the processes of phase transformations, as well as thermal expansion; (4) resistance to radiation damage and their accumulation during operation [4,5]. Additionally, an important factor in the development of new types of nuclear fuel is given to the possibility of replacing traditional uranium fuel with plutonium, the interest in which lies in the possibility of eliminating the accumulation of transuranium elements, uranium decay products, and radioactive waste [6,7,8]. The use of plutonium fuel, in turn, makes it possible to eliminate a number of problems, including those associated with the accumulation of decay products and transuranium elements [9,10].

One of the types of new fuel is fuel elements based on dispersed nuclear fuel [11,12]. This fuel is based on the possibility of using plutonium placed in an inert matrix, which makes it possible to remove part of the heat, as well as absorb most of the radiation damage caused both by nuclear reactions with neutrons and by the resulting decay products [13,14,15]. Inert matrices are usually based on oxide ceramics or microparticles, such compounds as ZrO_2_, MgO, CeO_2_, BeO, Al_2_O_3_, etc. [16,17,18,19,20]. The choice of these oxide ceramics is due to their resistance to external influences, as well as physicochemical and structural characteristics. Thus, for example, in a number of works it was shown that the use of oxide ceramic microparticles makes it possible to reduce the degree of radiation damage, and the obtained results of the kinetics of radiation defects and their accumulation make it possible to predict the scope and potential service life [21,22].

One of the promising materials in this direction of using them as inert matrix materials are zirconium dioxide microparticles, which have high mechanical strength and insulating characteristics [23,24,25]. However, according to a number of experimental studies, one of the disadvantages of these structures is their low resistance to phase polymorphic transformations under the action of irradiation, which negatively affects their strength and thermal parameters. One of the ways to increase the resistance of ZrO_2_ ceramics to radiation damage, as well as to mechanical softening as a result of external influences, is doping them with oxide compounds that have protective or reinforcing properties, the combination of which makes it possible to increase the strength of ZrO_2_, and the possibility of obtaining two-phase ceramics by nanosized particles opens up prospects in area of inert matrices and their application [26,27,28]. Interest in nanosized ceramics is also due to the possibility of increasing strength by changing the dislocation density, as well as to interboundary effects associated with the presence of several phases in the structure. The most promising dopants among oxide ceramics are cerium oxide (CeO_2_) or magnesium oxide (MgO), which have excellent properties, and in combination with the properties of ZrO_2_, it makes it possible to create new two-phase ceramics with high resistance to external influences [29,30].

Based on this, the purpose of this work is to study the properties of ZrO_2_-CeO_2_ composite ceramics depending on the content of the oxide components, as well as the effect of varying the components on the strength properties of the synthesized ceramics. The choice of CeO_2_ as a dopant is due to its protective properties, which provide an increase in the resistance of composite ceramics to external influences, and also have a significant effect on the strength properties. At the same time, as was shown in a number of works [31,32,33], the use of CeO_2_ as a dopant makes it possible to increase not only the strength properties, but also significantly change the resistance to radiation damage, since this material has an increased resistance to radiation embrittlement and swelling. Additionally, the protective properties of CeO_2_ will make it possible in the future to reduce the rate of polymorphic transformations in zirconium dioxide, which, as is known from a number of works [34,35], are characteristic of this type of ceramics under radiation exposure.

## 2. Experimental Part

To obtain (1 − x)ZrO_2_ − xCeO_2_, Sigma Aldrich (St. Louis, MO, USA) ZrO_2_ and CeO_2_ powders were used, which have a chemical purity of 99.95% and grain sizes of no more than 1 μm. The x variation was 0.05, 0.10, 0.15, 0.25, and 0.50. Synthesis of (1 − x)ZrO_2_ − xCeO_2_ composite ceramics was carried out in two stages. The initial mixtures were weighed in certain ratios with a total mass of the mixture of 10 g, which was subsequently subjected to mixing.

At the first stage, the selected powders were weighed, followed by mechanochemical grinding in a PULVERISETTE 6 planetary mill (Fritsch international, Idar-Oberstein, Germany) in a tungsten carbide cup for 1 h at a grinding speed of 400 rpm. After grinding, the resulting mixtures were subjected to dispersion and separation into portions for further thermal annealing. At the same time, the analysis of the obtained mixtures showed that they do not contain any impurities associated with the processes of mixing in a glass of tungsten carbide.

The second stage consisted in thermal sintering of the obtained samples at a temperature of 1500 °C for 1 h in a SNOL muffle furnace, at a heating rate of 10 °C/min and a cooling time of 24 h to reach room temperature. 

The study of phase transformations depending on the content of the components was investigated using the method of X-ray phase analysis by taking diffraction patterns in the range of 2θ = 20–90° and subsequent interpretation of the observed changes using the PDF-2 (2016) database. X-ray diffraction patterns were taken on a D8 Advance ECO X-ray diffractometer (Bruker, Germany). To analyze the structural parameters, as well as their changes, the Diffrac EVA v.4.2 program code was used. 

The study of the strength properties of ceramics was investigated using the indentation method to determine the hardness values of ceramics and their changes depending on the phase composition, as well as the single compression method to determine the resistance to cracking.

## 3. Results and Discussion

**Figure 1:**
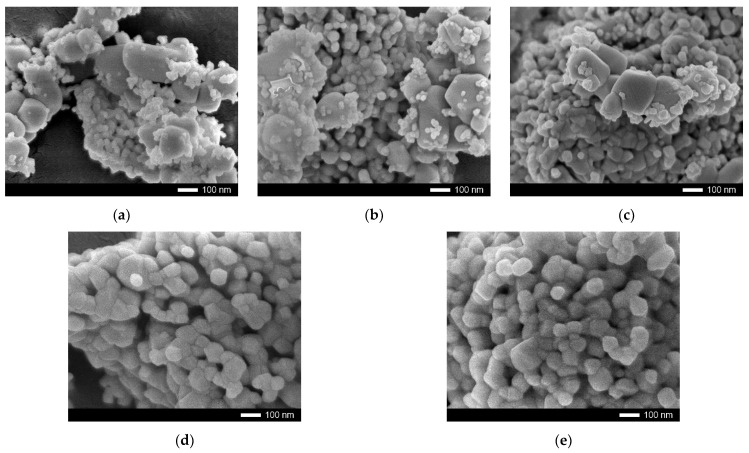

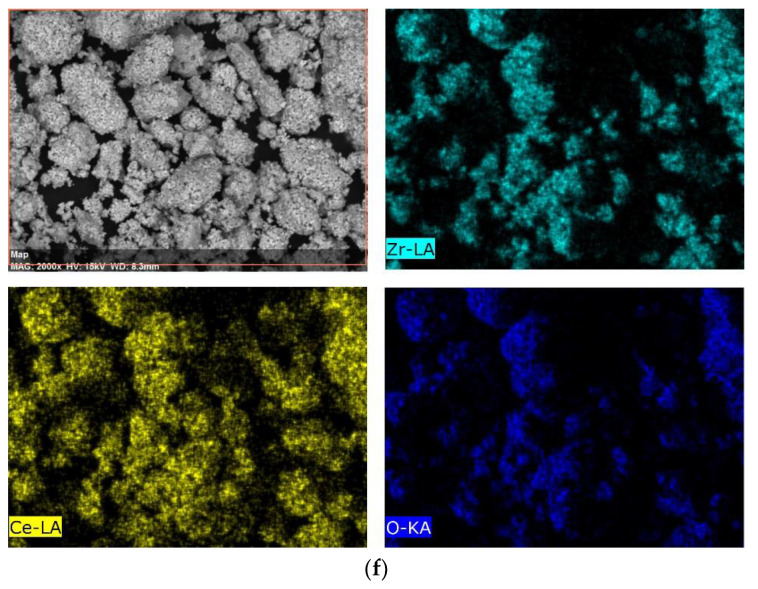
The results of SEM images of (1 − x)ZrO_2_ − xCeO_2_ ceramic grains depending on the concentration of CeO_2_ in the composition: (**a**) 0.05; (**b**) 0.10; (**c**) 0.15; (**d**) 0.25; (**e**) 0.50; (**f**) mapping results of the 0.5 ZrO_2_–0.5 CeO_2_ sample reflecting the isotropic distribution of zirconium and cerium in the structure of ceramics.

Figure 1 shows the results of the analysis of morphological features of synthesized (1 − x)ZrO_2_ − xCeO_2_ ceramics depending on the concentration of CeO_2_ in the composition.

As can be seen from the data presented, a change in the composition of the ceramic components leads to an increase in the degree of grain size homogeneity, as well as their decrease. At the same time, for samples with a CeO_2_ concentration of 0.05–0.15, there are clear differences in grain size and shape, which indicates that the ceramic structure is a mixture of two phases with different properties.

**Figure 2:**
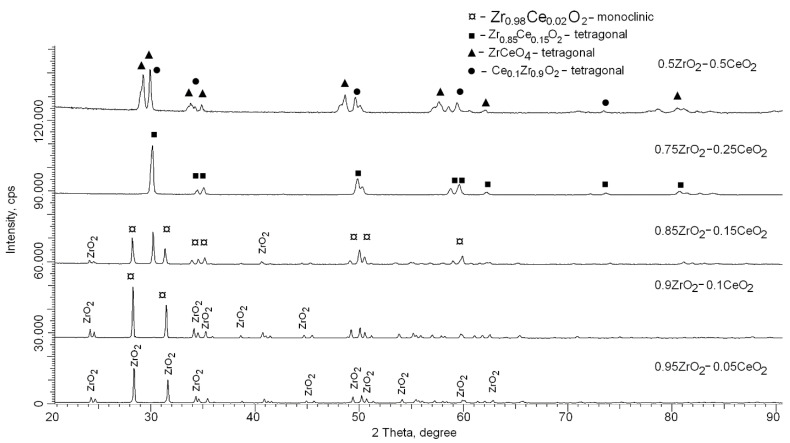
X-ray diffraction patterns of the studied samples depending on the content of the components.

Figure 2 shows X-ray diffraction patterns of the studied samples of synthesized (1 − x)ZrO_2_ − xCeO_2_ composite ceramics with a variation of the x component. The presented diffraction patterns are typical for polycrystalline well-crystallized samples, and the observed changes in diffraction lines indicate that phase transformations are observed in the composition of ceramics depending on the concentration of the components.

In the case of a CeO_2_ dopant content of 0.05, the main diffraction reflections observed in the diffraction pattern correspond to the ZrO_2_ monoclinic phase, with the spatial syngony P21/c(14). At the same time, the shape of diffraction reflections, as well as the ratio of the areas of diffraction reflections and background radiation, which reflects the degree of structural ordering, which amounted to more than 89%, indicates a rather high crystallinity degree of the synthesized samples. Additionally, in this case, the presence of any low-intensity reflections characteristic of other phases, including the CeO_2_ phase or substitution-type phases, is not observed. An analysis of the structural parameters for the ZrO_2_ phase showed that the crystal lattice parameters were a = 5.10953 Å, b = 5.18829 Å, c = 5.29281 Å, β = 98.950°, V = 138.6 Å^3^. At the same time, the differences in the crystal lattice parameters of the experimentally obtained and card values from the database (PDF-01-070-2491) may be due to mechanical deformation processes associated with mechanochemical mixing and subsequent thermal annealing.

With an increase in the CeO_2_ component concentration from 0.05 to 0.1, a shift in the main reflections characteristic of the Zr_0.98_Ce_0.02_O_2_ type substitution phase with a monoclinic type of crystal lattice and the P21/c(14) spatial syngony, the same as for the ZrO_2_ phase, is observed in the structure of the synthesized ceramics. At the same time, the formation of this phase is due to the processes of partial replacement of zirconium ions by cerium ions, followed by phase transformation. Additionally, a detailed analysis of these samples revealed the presence of low-intensity reflections characteristic of the ZrO_2_ tetragonal phase, the content of which is no more than 6% of the total structure. The presence of reflections characteristic of the tetragonal phase indicates that the addition of cerium oxide in small amounts initiates not only the processes associated with the formation of a substitution phase of the Zr_0.98_Ce_0.02_O_2_ type, but also the processes of polymorphic transformations of the Monoclinic-ZrO_2_ → Tetragonal-ZrO_2_ type. The occurrence of polymorphic transformations of this type may be due to the partial replacement of zirconium ions by cerium ions, which leads to an increase in the number of vacancies and free zirconium ions, the presence of which makes it possible to initiate the formation of the ZrO_2_ tetragonal structure.

In the case of samples with a concentration of CeO_2_ equal to 0.15 in the composition of the initial ceramics, no significant changes in the form of the formation of new phases were found, whereas the main processes of phase transformations are associated primarily with an increase in the ZrO_2_ phase with a tetragonal type of crystal lattice, the content of which was more than 30%.

With an increase in the concentration of CeO_2_ from 0.15 to 0.25, another process of phase transformations is observed, combined with a polymorphic transformation of the monoclinic type of the crystal structure into a tetragonal one. At the same time, the main phase dominating in the composition of ceramics with a CeO_2_ concentration of 0.25 is the tetragonal Zr_0.85_Ce_0.15_O_2_ substitution phase with the P42/nmc(137) spatial system. The formation of this phase is due to the presence of a high content of CeO_2_ in the composition of the initial mixture subjected to mechanical mixing and subsequent thermal annealing, which, as was shown earlier for lower concentrations, leads to the formation of substitution phases and polymorphic transformations of the Monoclinic-ZrO_2_ → Tetragonal-ZrO_2_ type. In this case, the processes of polymorphic transformations due to the presence of a large amount of cerium in the structure are also accompanied by substitution processes with subsequent formation of the Zr_0.85_Ce_0.15_O_2_ phase. At a CeO_2_ concentration of 0.5, two tetragonal phases ZrCeO_4_ and Ce_0.1_Zr_0.9_O_2_ are observed in the structure.

**Figure 3:**
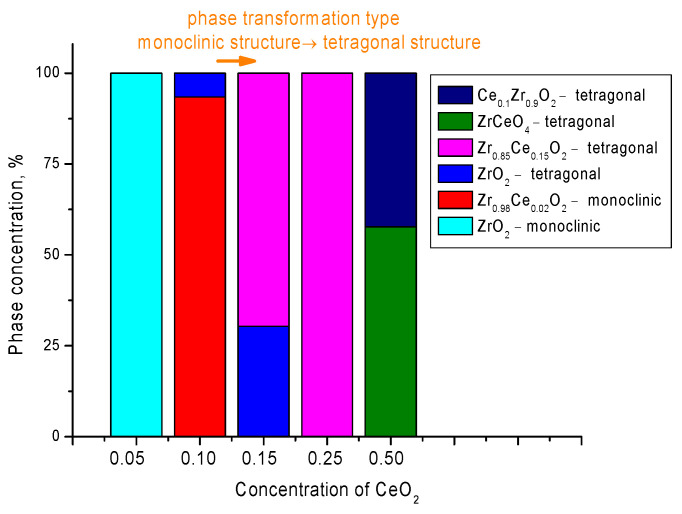
Phase composition diagram of synthesized (1 – x)ZrO_2_ − xCeO_2_ ceramics.

Figure 3 shows the diagram of phase transformations and the percentage of all established phases depending on the concentration of CeO_2_ in the composition of ceramics. The change in the crystal structure with an increase in the concentration of CeO_2_ is primarily due to the processes of partial replacement of zirconium ions by cerium ions, which leads to the formation of phases characteristic of structures similar to substitutional solid solutions with a nonstoichiometric Zr/Ce ratio in the composition. Secondly, an increase in the concentration of CeO_2_ in the structure at high concentrations of 0.25–0.5 leads to the formation of complex oxides of the ZrCeO_4_ type, as well as a transformation of the monoclinic → tetragonal structure type.

As can be seen from the data presented in the phase composition diagram, the processes of phase transformations of the monoclinic → tetragonal structure type can be divided into the following stages depending on the concentration of CeO_2_ in the composition of ceramics. The first stage is characterized by the nucleation of the ZrO_2_ tetragonal phase at a CeO_2_ content of 0.1; at the same time, the formation of this phase is also accompanied by the dominance of the Zr_0.98_Ce_0.02_O_2_ substitution phase with a monoclinic type of crystal lattice. With an increase in the CeO_2_ concentration, the formation of the Zr_0.85_Ce_0.15_O_2_ phase is observed, which, at a CeO_2_ concentration of 0.20, leads to the formation of a single-phase ceramic with a tetragonal crystal structure. With an increase in the CeO_2_ concentration to 0.25, the formation of two phases ZrCeO_4_ and Ce_0.1_Zr_0.9_O_2_ with a tetragonal type of crystal lattice is observed in the ceramic structure, the content of which is approximately equal. Based on the obtained diffraction patterns, the following phase transformations were established depending on the concentration of the components in ceramics: Monoclinic-ZrO_2_ → Monoclinic − Zr_0.98_Ce_0.02_O_2_/Tetragonal – ZrO_2_ → Tetragonal − Zr_0.85_Ce_0.15_O_2_ → Tetragonal − ZrCeO_4_/Ce_0.1_Zr_0.9_O_2_.

Table 1 presents the results of changing the crystal lattice parameters depending on the phase composition of the synthesized (1 − x)ZrO_2_ − xCeO_2_ ceramics.

As can be seen from the data presented, a change in the phase composition of ceramics leads to an increase in the crystal lattice parameters, which is typical for processes associated with phase transformations and substitution effects, leading to the formation of new phases.

**Figure 4:**
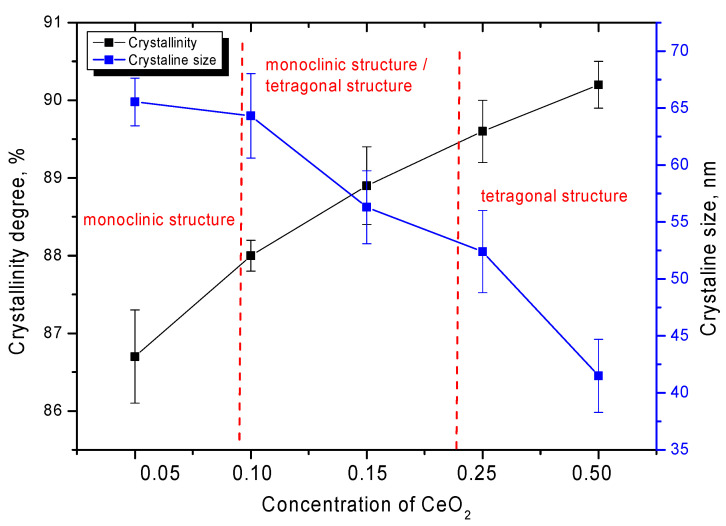
Dependences of the change in the crystallinity degree and crystallite sizes on the CeO_2_ concentration in the composition of ceramics.

A change in the phase composition, accompanied not only by phase transformations, but also by subsequent transformations of the monoclinic structure → tetragonal structure type, leads to a change in the degree of crystallinity or structural ordering of the samples under study. Figure 4 shows the results of the change in the crystallinity degree, as well as the size of crystallites, which were estimated from the data of X-ray diffraction patterns using the Scherer equation.

As can be seen from the data presented, phase transformations lead to an increase in the structural ordering degree, whereas a change in the size of crystallites is also observed, which can be due to processes associated with the rearrangement of the crystal structure as a result of a change in the phase composition of ceramics. At the same time, a decrease in the size of crystallites with a change in the phase composition leads to a change in the dislocation density in the structure of ceramics, an increase in which can have a significant effect on the strength properties of ceramics [28,29]. As it is known, a change in the dislocation density can cause the so-called effect of dislocation hardening, due to a decrease in the probability of propagation of microcracks that occur under external influences. This effect is used quite often in metallurgy, to create high-strength materials that are subject to external influences and shocks. In the case of inert matrix materials, high strength and crack resistance are among the leading indicators that determine their service life, since during operation, ceramics can be subjected not only to radiation exposure, one of the effects of which is swelling of materials, but also to thermal heating, as well as mechanical shocks or pressures that can lead to deformation and cracking of ceramic materials.

**Figure 5:**
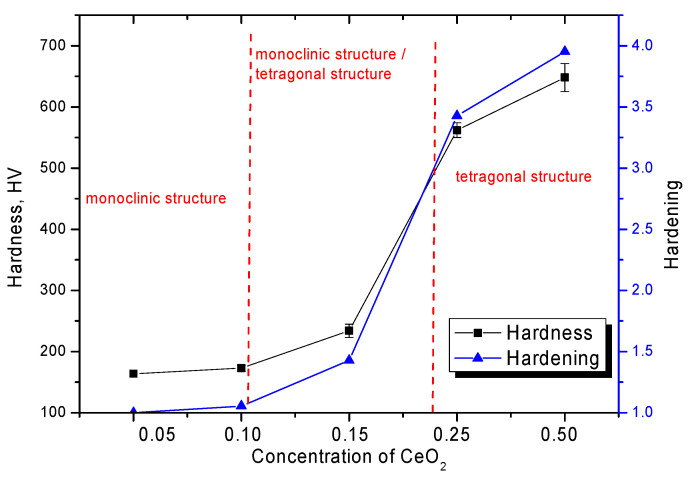
Results of the change in the hardness of ceramics depending on the CeO_2_ concentration.

**Figure 6:**
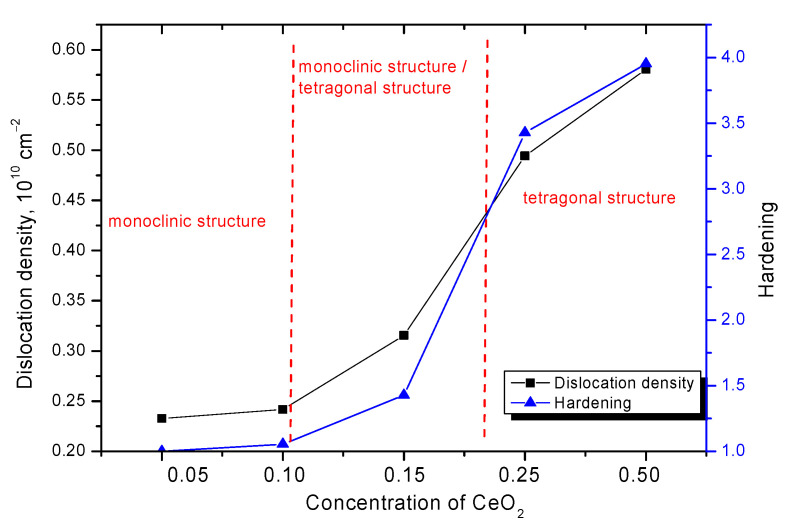
Dependence of the change in the dislocation density and the hardening degree of ceramics on the CeO_2_ concentration in the composition of ceramics.

**Figure 7:**
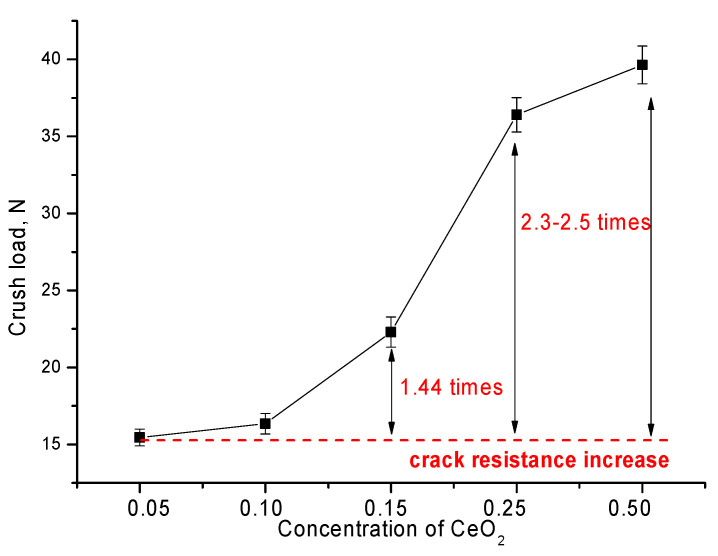
Graph of the change in crack resistance during a single compression.

Figure 5, Figure 6 and Figure 7 show the results of the change in strength characteristics, as well as the dependence of the influence of dislocation density on hardening and increase in crack resistance.

The general trend in the change in strength characteristics can be divided into three characteristic areas that have a pronounced dependence on the phase composition of the synthesized ceramics. In the case when the ZrO_2_ phase of the monoclinic type dominates in the ceramic structure, the hardness and crack resistance values are quite low. At the same time, the formation of the Zr_0.98_Ce_0.02_O_2_ phase in the structure of ceramics does not lead to significant changes in the strength characteristics. The increase in hardness and crack resistance in this case was no more than 3–5%, which indicates a small effect of the change in the phase composition during the replacement phase formation on the strength characteristics. The formation in the structure and the subsequent increase in the contribution of ZrO_2_ tetragonal phases leads to a sharp increase in strength characteristics, which indicates the strengthening of ceramics due to phase transformations. Additionally, an important role in this process is played by the change in dislocation density, the change of which, as the data in Figure 6 shows, has a good correlation with the change in strength properties. In the case when monoclinic phases are not observed in the structure of ceramics at high CeO_2_ concentrations, the increase in strength and crack resistance is more than 3.5–4 and 2.3–2.5 times, respectively, compared with samples in which the monoclinic type of crystal lattice dominates. At the same time, the change in the dislocation density depending on the phase composition has a similar pattern of changes as the strength properties, which indicates that the hardening of ceramics is affected not only by a change in the phase composition, but also by a change in the dislocation density.

Hardening mechanisms can be explained as follows. A change in the dislocation density due to a decrease in the size of crystallites leads to the formation of additional obstacles in the form of grain boundaries or dislocation loops, to the propagation of microcracks under the appearance of external pressures or stresses. At the same time, an important role in changing the dislocation density is played by a change in the phase composition and phase transformations of the crystal structure from monoclinic to tetragonal. In the case of a monoclinic structure, the crystallite sizes are quite large, and from the assessment of the degree of structural ordering in the composition of ceramics, a sufficient number of disordered regions and defective inclusions are observed, the presence of which leads to accelerated destruction under external influences. The presence of a tetragonal phase in the composition leads to an increase in hardening, as well as to fragmentation of crystallites, which leads to an increase in the dislocation density, an increase in which, as can be seen from the data presented, leads to the appearance of the dislocation hardening effect.

## 4. Conclusions

Thus, the following conclusions can be drawn.

During the studies performed, it was found that a change in the CeO_2_ concentration in the composition of ceramics leads to the formation in the structure of first substitution phases of the Zr_0.98_Ce_0.02_O_2_ type of the monoclinic type, then the tetragonal Zr_0.85_Ce_0.15_O_2_ phase, the appearance of which is also accompanied by an increase in the contribution of polymorphic transformations of the Monoclinic-ZrO_2_ → Tetragonal-ZrO_2_ type. At CeO_2_ concentrations above 0.15, a phase transformation of the structure of the type monoclinic structure → tetragonal structure is observed with dominance in the structure of substitutional phases of the Zr_0.85_Ce_0.15_O_2_ and Ce_0.1_Zr_0.9_O_2_ types.

When analyzing the effect of the phase composition on the strength properties of ceramics, it was found that the displacement of the ZrO_2_ phase from the ceramic structure with its subsequent transformation into the Zr_0.85_Ce_0.15_O_2_ or Ce_0.1_Zr_0.9_O_2_ phase leads to the strengthening of the ceramics by more than 3.5–4 times. The results of resistance to cracking under single compression showed that the formation of the ZrCeO_4_ phase in the structure of ceramics leads to an increase in the resistance of ceramics to cracking by more than 2.5 times.

Further prospects of these studies will be directed to the study of the resistance of these ceramics to thermal aging, as well as the assessment of resistance to external influences, including radiation damage caused by heavy ion irradiation.

## Figures and Tables

**Table 1:** Data on the crystal lattice parameters of the studied ceramic samples (the parameters were determined using the Rietveld method).

Lattice Parameter	Concentration of CeO_2_				
Phase	0.05	0.10	0.15	0.25	0.50
ZrO_2_—monoclinic P21/c(14)	a = 5.10953 ± 0.00012 Å, b = 5.18829 ± 0.00015 Å, c = 5.29281 ± 0.00016 Å, β = 98.950°	-	-	-	-
Zr_0.98_Ce_0.02_O_2_—monoclinic P21/c(14)	-	a = 5.14495 ± 0.00021 Å, b = 5.20584 ± 0.00016 Å, c = 5.30924 ± 0.00017 Å, β = 98,852°	a = 5.15503 ± 0.00015 Å, b = 5.20990 ± 0.00027 Å, c = 5.30404 ± 0.00023 Å, β = 98.773°	-	-
ZrO_2_—tetragonal P42/nmc(137)	-	a = 3.58742 ± 0.00015 Å, c = 5.17585 ± 0.00017 Å	a = 3.59234 ± 0.00011 Å, c = 5.18498 ± 0.00016 Å	-	-
Zr_0.85_Ce_0.15_O_2_—tetragonal, P42/nmc(137)	-	-	-	a = 3.61188 ± 0.00026 Å, c = 5.18693 ± 0.00017 Å	-
ZrCeO_4_—tetragonal P42/nmc(137)	-	-	-	-	a = 3.73690 ± 0.00017 Å, c = 5.29911 ± 0.00012 Å
Ce_0.1_Zr_0.9_O_2_—tetragonal P42/nmc(137)	-	-	-	-	a = 3.62745 ± 0.00014 Å, c = 5.22967 ± 0.00018 Å

## Data Availability

Not applicable.
